# Recruitment of the premotor cortex during arithmetic operations by the monkey

**DOI:** 10.1038/s41598-024-56755-2

**Published:** 2024-03-28

**Authors:** Sumito Okuyama, Toshinobu Kuki, Hajime Mushiake

**Affiliations:** 1 Department of Physiology, Tohoku University School of Medicine, Sendai, 980-8575 Japan; 2 Department of Neurosurgery, Southern Tohoku General Hospital, Miyagi, 989-2483 Japan

**Keywords:** Cognitive neuroscience, Sensorimotor processing, Premotor cortex

## Abstract

Arithmetic operations are complex mental processes rooted in the abstract concept of numerosity. Despite the significance, the neural architecture responsible for these operations has remained largely uncharted. In this study, we explored the presence of specific neuronal activity in the dorsal premotor cortex of the monkey dedicated to numerical addition and subtraction. Our findings reveal that many of these neural activities undergo a transformation, shifting their coding from arithmetic to motor representations. These motor representations include information about which hand to use and the number of steps involved in the action. We consistently observed that cells related to the right-hand encoded addition, while those linked to the left-hand encoded subtraction, suggesting that arithmetic operations and motor commands are intertwining with each other. Furthermore, we used a multivariate decoding technique to predict the monkey’s behaviour based on the activity of these arithmetic-related cells. The classifier trained to discern arithmetic operations, including addition and subtraction, not only predicted the arithmetic decisions but also the subsequent motor actions of the right and left-hand. These findings imply a cognitive extension of the motor cortex’s function, where inherent neural systems are repurposed to facilitate arithmetic operations.

## Introduction

There is some evidence that addition and subtraction, fundamental arithmetic operations deeply ingrained in our daily lives, can be executed nonverbally. An indigenous Amazonian group, despite lacking a numerical notation system beyond 5, exhibited a capacity to comprehend numerical addition and subtraction in a manner similar to French speakers^1^. Even infants without explicit numerical knowledge displayed surprise when faced with incorrect outcomes of addition and subtraction^2,3^. This ability is not exclusive to humans; various nonhuman primates, including chimpanzees^4^, orangutans^5^, rhesus monkeys^6,7^, lemurs^8^, and vervet monkeys^9^, have also demonstrated competence in these operations, suggesting the existence of nonverbal numerical operation systems.

However, previous investigations into nonverbal arithmetic have raised questions. The neural coding of addition and subtraction remains uncertain. Previous neurophysiological studies of nonhuman primates have indicated that the ventral intraparietal area in the parietal cortex^10–12^ and the prefrontal cortex (PFC)^13,14^ are related to numerosity. Human functional neuroimaging studies have shown that the intraparietal sulcus and PFC are activated during symbolic^15–19^ and nonsymbolic arithmetic tasks^20,21^, indicating that they may be the main hubs for arithmetic operations^22^. However, other candidates are emerging. A recent report proposed that single neurons in the human medial temporal lobe play a role in processing arithmetic rules^23^. Yet, the exact neural circuitry underlying arithmetic operations remains unidentified. Human resting-state functional connectivity studies have revealed arithmetic-related connectivity between the parietal and premotor cortices^21,24^ which predicted the performance of children in a math test^25^. One possible candidate area for numerical operations is the dorsal premotor cortex (PMd), recognized for guiding voluntary arm movements. Mushiake et al*.* demonstrated the role of PMd in visually guided and memory-guided behavioural rules for sequential motor actions^26^. The guidance is far more arbitrary and flexible. PMd encodes abstract action plan^27^, abstract rule^28,29^, numerical rule^30^ and distance in rank^31,32^. Notably, sensorimotor mapping in the PMd is indirectly determined by contextual conditions^33^. PMd receives robust input from the superior parietal cortex^34,35^, which in turn is implicated in numerical representations of actions^36,37^ and receives input from the ventral intraparietal cortex^38,39^, the hub of numerosity representation. Consequently, PMd is a plausible candidate for representing numerical operations, including addition and subtraction.

Further inquiry concerns how these operations manifest at the cellular level. One hypothesis proposes the existence of specialized cells dedicated to encoding arithmetic operations. However, in the parietal cortex, sensorimotor circuits, which initially evolved for basic functions, may have been repurposed for advanced cognitive tasks such as mental arithmetic^20,40^. Alternatively, motor-related cells may have been adapted for arithmetic tasks, effectively integrating arithmetic into motor functions because of limited neural resources in nonhuman primates. It is plausible that existing neural frameworks in the motor cortex are involved in addition and subtraction.

To address these issues, we constructed tasks separating motor and arithmetic aspects, and investigated cellular discharge within the PMd.

## Results

### Behaviour

**Figure 1:**
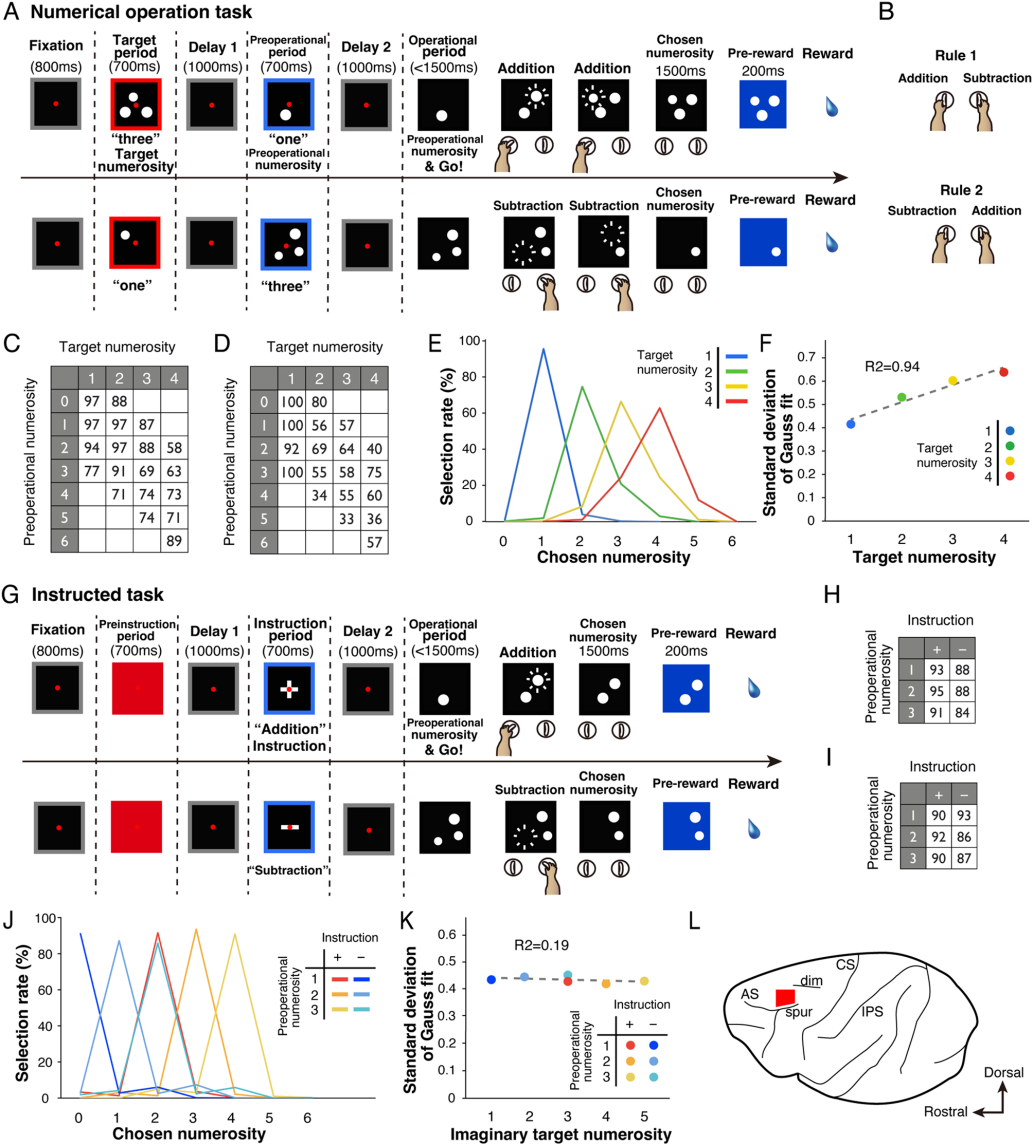
Schematic illustration of the numerical operation tasks and behavioural performance. (**A**) The time paradigm of the numerical operation task. After the monkeys gazed at a fixation point, the target numerosity in a red square was presented. After Delay 1, the preoperational numerosity was shown in a blue square. After Delay 2, a Go signal allowed the monkeys to manipulate the preoperational numerosity to match the remembered target numerosity using manipulanda held in both hands. If the monkeys remained without manipulation, the presented numerosity was recorded as their decision. In case the chosen numerosity matched the target numerosity, they were rewarded with juice. (**B**) The relationship between manipulanda (left vs. right) and operations (addition vs. subtraction) was assessed using two rules. (**C**,**D**) Nineteen numerical pairs were prepared during recordings. Average selection rates are shown according to the target numerosity (chance level = 25%). (**E**) Mean selection rates of the chosen numerosity for the two monkeys. (**F**) Standard deviations of Gaussian fits for the monkeys. The dashed line represents the best-fit linear model. (**G**) Time course of the Instructed task. After the monkeys fixated on a red circle, pre-instruction was presented through a red square alerting the monkeys of the second task. Following Delay 1, an instruction of addition or subtraction appeared in a blue square. The monkeys were required to manipulate the preoperational numerosity according to the remembered arithmetic instruction. (**H**,**I**) Three preoperational numerosities were prepared for each instruction. The mean rate of correct responses for each monkey is shown. (**J**) Average selection rates of the chosen numerosity by the two monkeys. (**K**) Standard deviations of a Gaussian fit plotted for each pair. (**L**) Cortical map of the recording site. Lateral view of the left hemisphere showing the extent of the surveyed area in a red rectangle. *AS* arcuate sulcus, *spur* spur of AS, *dim* superior precentral dimple, *CS* central sulcus, *IPS* intraparietal sulcus.

Two monkeys were trained to perform numerical operation tasks (Fig. 1A,G) (see “Methods” for details). These tasks involved two components: a numerical operation task and an instructed task. In the numerical operation task (Fig. 1A), the monkeys were tasked with manipulating the second numerosity (preoperational numerosity) to match their memory of the first numerosity (target numerosity) using manipulanda held in both hands. After a Go signal, the monkeys were allowed to perform numerical manipulations through incremental addition or subtraction. If they paused for 1.5 s, the displayed numerosity was recorded as their decision (chosen numerosity). If the chosen numerosity matched the target numerosity, they received a reward. Two manipulation rules were in place (Fig. 1B): in Rule 1, the left manipulanda corresponded to addition, and the right manipulanda to subtraction; while in Rule 2, these relationships were reversed. The target numerosity and preoperational numerosity ranged from 1 to 4 and 0 to 6, respectively, followed by delay periods (Fig. 1C,D). Both monkeys performed significantly better than chance for each target numerosity (Fig. 1E, binomial test, *P* < 0.01). Similar to previous observations^12,41^, the monkeys’ performance declined and their decisions became more variable with increasing target numerosity (numerical size effect) (Fig. 1F, linear regression, *r*^2^ = 0.94%, *P* < 0.05), indicating that the monkeys executed numerosity-based operations in this task.

In the second task, monkeys were tasked with performing instructed operations regardless of preoperational numerosity (Fig. 1G; see “Methods”). The preoperational numerosity varied randomly from 1 to 3 for each instruction (Fig. 1H,I). Again, the monkeys’ performance exceeded chance levels (Fig. 1J). However, unlike the first task, there was no observed numerical size effect in the second task (Fig. 1K, linear regression, *r*^2^ = 0.19%, *P* = 0.39), indicating that in the instructed task, the monkeys executed instructed operations without numerical influence.

In both tasks, monkeys that performed an inappropriate operation in the first step flexibly used another device (switching behaviour) to perform the expected numerical operation within a trial (Fig. S2). If the monkeys were responding in accordance with stimulus–response associations, they could not perform switching behaviour without reward information. The monkeys monitored the outcomes of operations with the intention to perform addition or subtraction; other strategies, such as ones based on stimulus–response associations, were abandoned.

To investigate whether the monkeys performed consistent operations across both tasks, we conducted a task switch from the numerical operation task to the instructed task. Remarkably, the monkeys’ initial operations remained unchanged before and after the task switch (Fig. S3, McNemar test, *P* = 1), suggesting that the monkeys executed consistent operations across both tasks.

### Arithmetic-related cells

**Figure 2:**
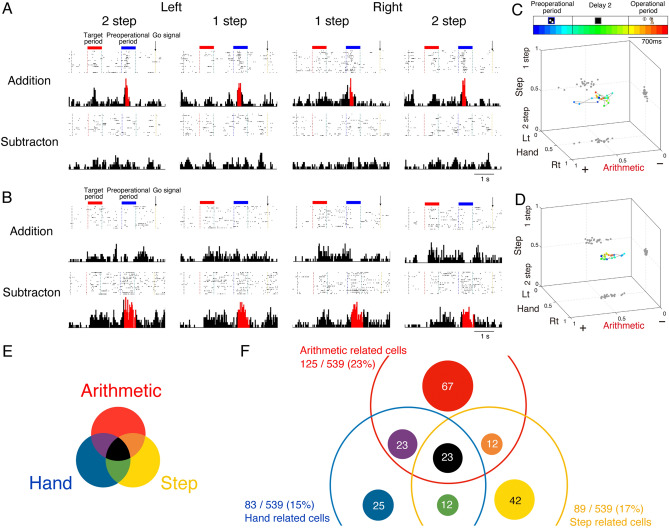
Two examples of selective PMd-cell activity for arithmetic operations in the numerical operation task and distribution of task related PMd cells. (**A**) Rasters and peri-event histograms showing cellular activity selective for addition tasks. (**B**) Subtraction-selective activity. (**C**) ROC values for each operation (arithmetic, hand, step) from the same cell shown in (**A**), representing the addition-related trajectory. The values start at the presentation of the preoperational numerosity and are separated from the adjacent values by 100 ms intervals. (**D**) Subtraction-related trajectory for the cell shown in (**B**). (**E**) The colours in each panel in (**A**), (**B**), and (**F**) correspond to the contrasting results from arithmetic-related (red), hand-related (blue), and step-related (yellow) activities. (**B**) Coloured circles illustrating the proportion of subgroups in arithmetic-related cells (middle), hand-related cells (left), and step-related cells (right). Subgroups were classified based on previous coding features during the preoperational numerosity period.

We conducted recordings from 539 cells in the left hemisphere’s PMd of a monkey performing two distinct numerical operation tasks (Fig. 1L). Initially, our focus was on neuronal activity during the preoperational period within the numerical operation task. A notable number of cells in the PMd exhibited variable activity patterns related to arithmetic operations. We begin by presenting clear examples of such arithmetic-related cells. As shown in Fig. 2A, we observed pronounced activities in a cell when the monkey was preparing for addition operations, irrespective of whether it used their left or right hand or executed one or two steps. Similarly, as shown in Fig. 2B, another displayed cell increased activity as the monkey prepared for subtraction operations. Hence, these two cells can be considered indicative of forthcoming arithmetic operations, either addition or subtraction.

To assess the extent to which PMd cells predict specific elements of numerical operations, we calculated receiver operating characteristic (ROC) statistics using continuous 100 ms time windows. These statistics involved three factors: arithmetic (addition vs. subtraction), hand (left hand vs. right hand), and step (1 step vs. 2 steps). ROC values, ranging from 0 to 1, allowed us to capture each cell’s preference for numerical operations (ROC values around 0.5 indicated no preference; values closer to 1 indicated a preference for addition, right hand, or 1 step; values closer to 0 indicated a preference for subtraction, left hand, or 2 steps). The plots for the first type of cells clearly shifted along the arithmetic axis, demonstrating their role in encoding arithmetic operations (Fig. 2C,D).

We conducted regression analyses every 100 ms during the preoperational period to identify significant factors related to the operation (arithmetic operation, hand movement, and number of steps). The dominant factors over this period were integrated and defined as the cell’s coding history. Cells were classified based on their coding history during the preoperational period, resulting in 197 out of the 539 PMd cells exhibiting arithmetic-related activities.

In the numerical operation task, the monkey had to compare two numerosities and decide on subsequent arithmetic operations. We considered the possibility that neural activities related to arithmetic operations might reflect the process of judging which numerosity is larger or smaller. To rule this out, we analysed neural activities during the instructed task, where the monkey was required to execute instructed operations without comparing numerosities. If cells exhibited similar relationships to arithmetic operations in both tasks, we deemed them to be related to arithmetic operation.

The neural activities and ROC analysis during the instructed task for the same cells shown in Fig. 2 revealed simple arithmetic-related features once again (Fig. S4A,B). However, 72 cells that exhibited arithmetic operation-related activities in the numerical operation task did not show differentiation based on the instruction in the instructed task. As a result, these cells were not classified as selectively related to arithmetic operations. Thus, out of the 539 PMd cells, 125 were selective to arithmetic operations, 83 were selective to hand movement, and 89 were selective to the number of steps, including overlapping preferences (Fig. 2E,F). Here we focused on the 125 arithmetic related cells.

**Figure 3:**
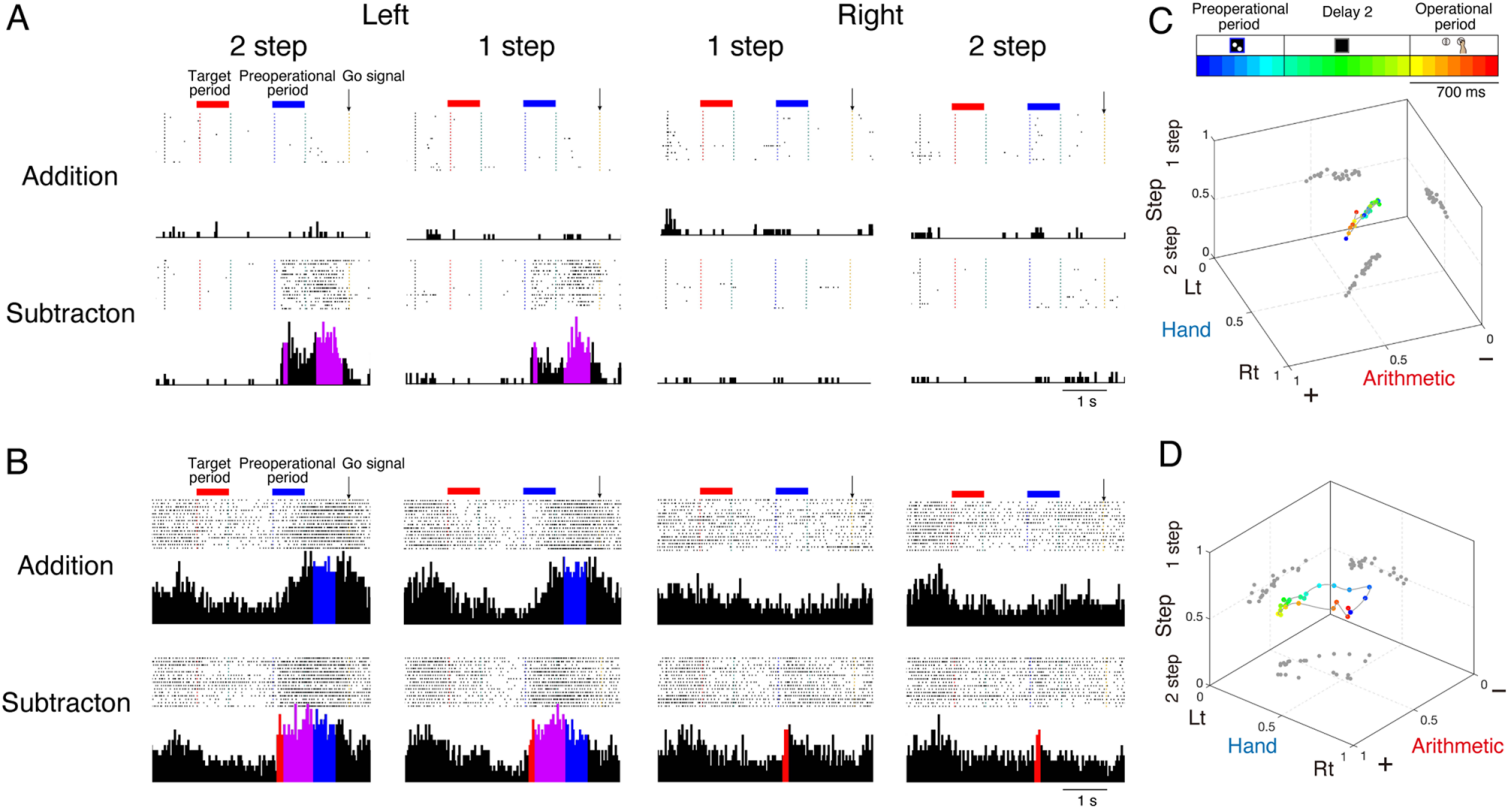
Arithmetic- and hand-related activity of PMd cells in the numerical operation task. (**A**) Rasters and peri-event histograms showing simultaneous selective cellular activity for arithmetic and hand operations. (**B**) Activity selection for arithmetic, both arithmetic and hand, and only hand operations. (**C**) ROC values for each operation (arithmetic, hand, and step) represent a combination of arithmetic- and hand-related trajectories from the cell shown in (**A**). (**D**) Trajectory for the cell shown in (**B**) transitioning from arithmetic to hand. Colours in the histogram correspond to those in Fig. 2E.

We also observed another type of neural activity in the following two cells, representing both arithmetic operations and hand movements (Fig. 3A,B). The cell shown in Fig. 3A simultaneously encoded both subtraction arithmetic operations and left-hand movements. By contrast, the cell shown in Fig. 3B shifted its representations from subtraction arithmetic operations to combinations of arithmetic and left-hand movements, and then to left-arm movements dynamically. The ROC value plots showed that these cells transitioned from encoding arithmetic operations to hand movements in distinct manners (Fig. 3C,D). The activities and ROC analysis in the instructed task for these cells are presented in Fig. S4C,D.

### Coding history of arithmetic-related cells

**Figure 4:**
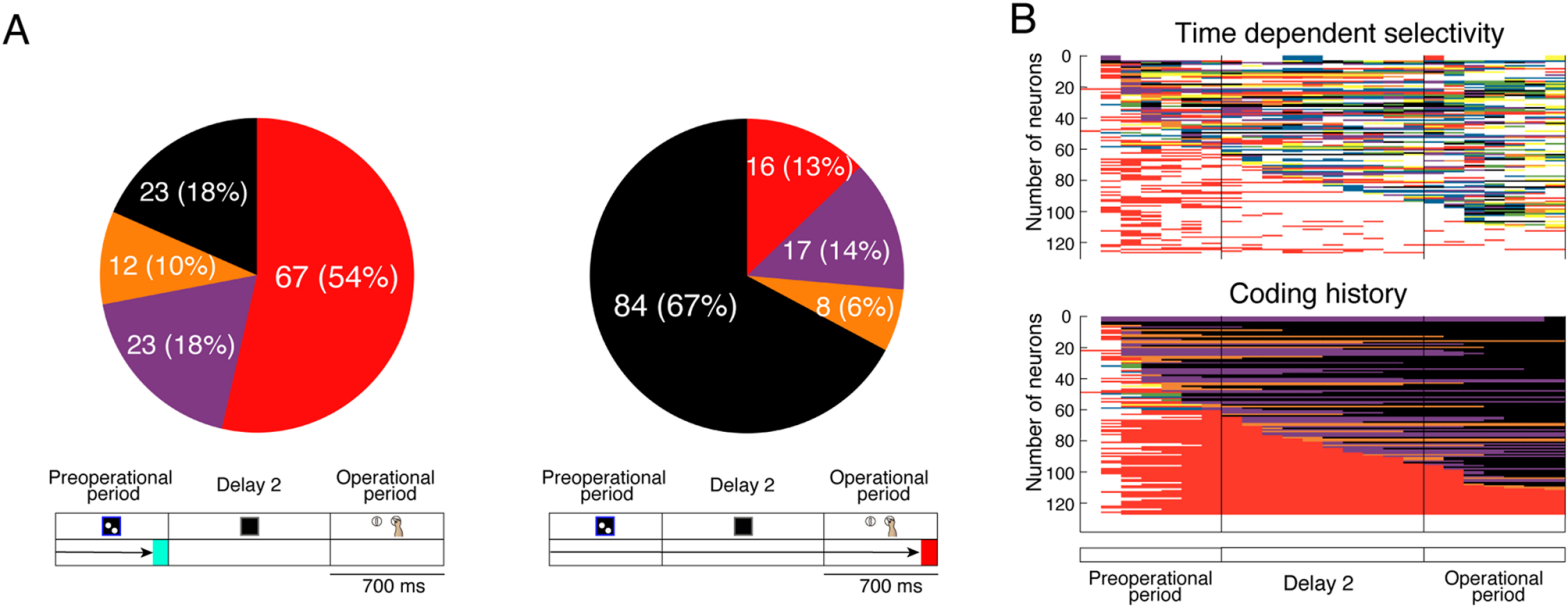
Change in the distribution of arithmetic-related PMd cells and coding history. Colours in panels (**A**) and (**B**) correspond with those in Fig. 2E. (**A**) Pie charts illustrating the proportion of subgroups in arithmetic-related cells up to the preoperational period (left) and the operational period (right). Subgroups were classified based on previous encoding features (coding history). (**B**) Time-dependent selectivity (upper) and temporal patterns of previous coding history (lower) for the 125 arithmetic-related cells are shown. Colours in each data correspond to those in Fig. 2E.

To investigate the temporal changes in response characteristics of the arithmetic cells, we analysed their coding history throughout both the preoperational and operational periods (Fig. 4). At the conclusion of the preoperational period, 67 of the 125 arithmetic cells exclusively encoded arithmetic operations (n = 67/125, 54%). Interestingly, by the end of the operational period, only 16 cells maintained exclusive coding for arithmetic (n = 16/125, 13%), indicating a significant reduction in purely arithmetic coding cells (Fig. 4A, McNemar test, *P* < 0.01). The majority of arithmetic cells did not exclusively code for arithmetic operations but changed their representations from arithmetic operations to hand or step information later in the task period (Fig. 4B).

### Dynamics of the arithmetic operation process

**Figure 5:**
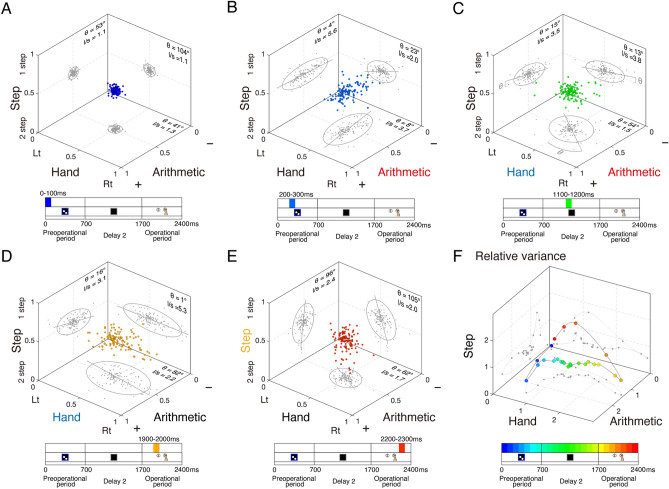
Dynamic response by a population of arithmetic cells in the numerical operation task. ROC analysis for each operation (arithmetic, hand, and step) in the 125 arithmetic-related cells. The analysis was performed for the following time windows: (**A**) 0–100 ms, (**B**) 200–300 ms, (**C**) 1100–1200 ms, (**D**) 1900–2000 ms, and (**E**) 2200–2300 ms after the onset of preoperational numerosity. A two-dimensional Gaussian fit was performed on the data projected to each plane. Ellipses represent the 95% confidence contour. θ, angle of ellipse’s long axis relative to axis of arithmetic or hand; l/s: ratio of length of the long to short axis. (**F**) Relative variances of each ROC value, calculated by normalizing to the average variance throughout the relevant period, are plotted.

Exploring the dynamics of the arithmetic operation process, we sought to understand how the 125 arithmetic cells discriminated between elements involved in numerical operations from the preoperational to operational period using ROC statistics for the three factors (Fig. 5). Each dataset was projected onto planes composed of two elements and fitted with an ellipse to visualise the distribution’s orientation. Immediately after the presentation of the preoperational numerosity, all plots were clustered around 0.5, and none of the elements were clearly distinguished (Fig. 5A). However, 200 ms later, the long axes of the ellipses aligned with the arithmetic operation axis, indicating a dependence on arithmetic operations (Fig. 5B). Subsequently, during the Delay 2 period, the plots expanded across a plane combining arithmetic and hand factors (Fig. 5C). During the operational period, the ellipses oriented themselves along the hand axis (Fig. 5D). Finally, in the latter part of the operational period, the distribution spread along the step axis (Fig. 5E). We normalized the variance of ROC values based on the mean variance across the entire period to demonstrate the relative change in variance over time (Fig. 5F). The increase in relative variance transitioned from arithmetic to hand and ultimately to step information. Consequently, it was observed that arithmetic-related cells initially coded arithmetic information at the population level and then dynamically shifted their coding content from hand information to step information over time. This transition from arithmetic coding to hand coding was also observed in the instructed task (Fig. S5: for the ROC analysis in the instructed task).

### Biased distribution of arithmetic- and hand-related cells

**Figure 6:**
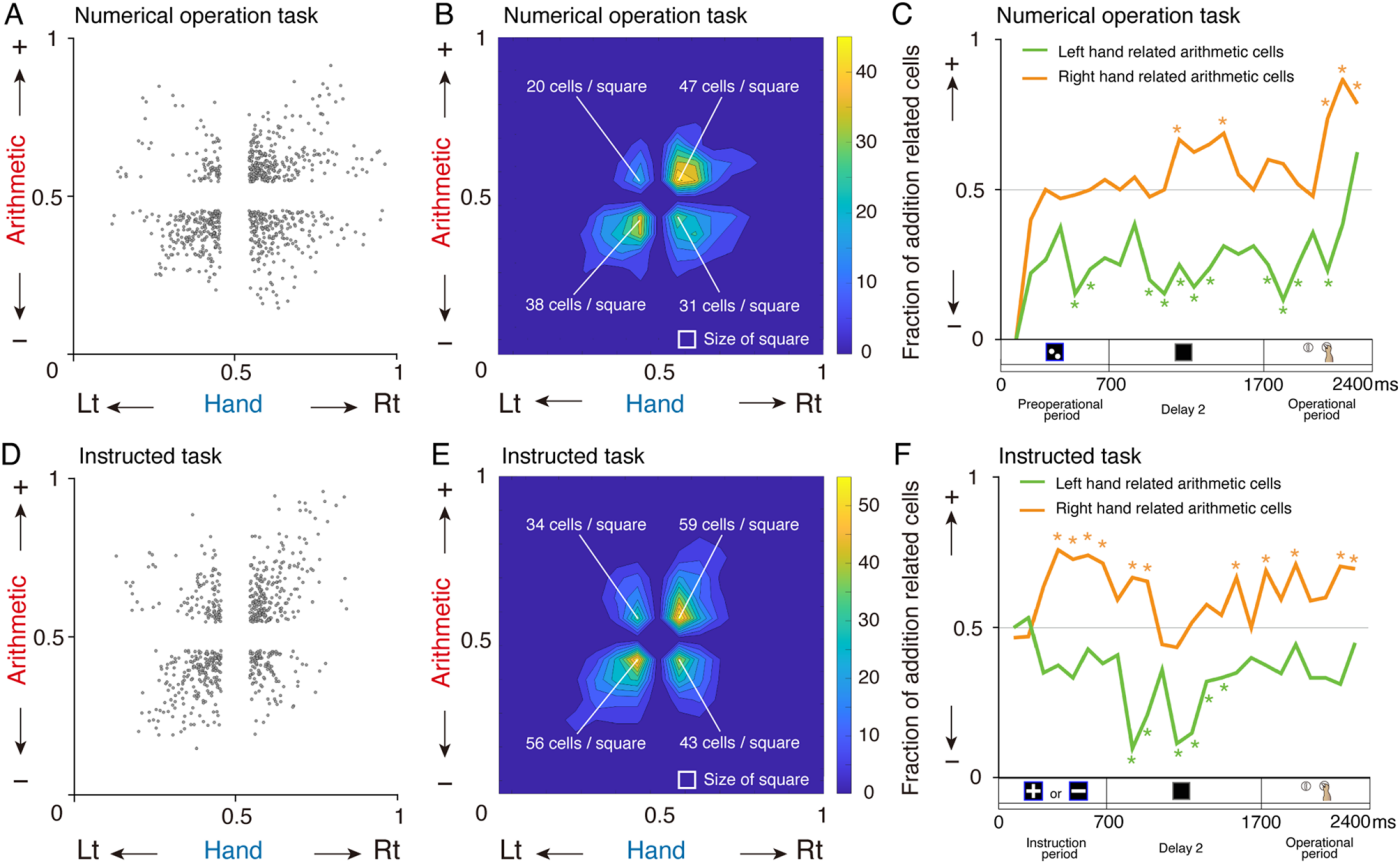
Distribution of simultaneous hand-encoding arithmetic cells. (**A**) Scatter plot representing ROC values of arithmetic-related cells showing significant differences between hand and arithmetic during the period of the numerical operation task. (**B**) Contour plot of the data obtained in (**A**). (**C**) Ratio of addition-related cells to hand-related arithmetic cells as a function of time in the numerical operation task. *P < 0.01, binomial test with *P*_chance_ = 0.05. (**D**–**F**) Results of the instructed task shown in the same format as in (**A**–**C**), respectively.

We identified several arithmetic cells that concurrently encoded arithmetic operations and hand movements (Fig. 3A). Subsequently, we investigated whether there was an imbalance in the number of cells corresponding to addition-subtraction operations and the left–right hands in those cells encoding both arithmetic and hand information simultaneously. We extracted ROC values from the arithmetic-related cells that exhibited a significant difference in both arithmetic and hand coding from the pre-operational to the operational period (permutation test, 1000 times, *P* < 0.05) (Fig. 6A). Figure 6B presents a contour plot representing the density of the ROC value distribution, revealing interesting biased distributions among quadrants corresponding to addition by the left-hand, subtraction by the left-hand, addition by the right hand, and subtraction by the right-hand. Figure 6C illustrates the fraction of addition-related cells distinguished by their hand associations throughout the trial period. We identified time windows that predominantly encoded addition or subtraction (binomial test with *P*_chance_ = 0.50, *P* < 0.05). Right-hand-related arithmetic cells more frequently encoded addition (5 statistically significant points in the time series; binomial test with *P*_chance_ = 0.05, *P* < 0.01). Conversely, left-hand-related arithmetic cells more frequently encoded subtraction (11 statistically significant points in the time series; binomial test, *P* < 0.01). This observation was consistent in the instructed task (Fig. 6D,E). Once again (Fig. 6F), right-hand-related cells more often encoded addition (11 statistically significant points in the time series; binomial test, *P* < 0.01), and left-hand-related cells more often encoded subtraction (6 statistically significant points in the time series; binomial test, *P* < 0.01). This relationship did not involve the process of comparing larger or smaller numerosities. Therefore, arithmetic cells simultaneously represent arithmetic operations and hand commands, with the right hand being more frequently associated with addition and the left hand more frequently associated with subtraction.

In a further analysis of the monkeys’ behaviour, we compared reaction time and performance between right- and left-hand responses (Fig. S6). Right-hand responses were faster in addition trials than in subtraction trials, whereas left-hand responses were faster in subtraction trials than in addition trials; performance did not differ between the hands. We surmised that the monkeys were monitoring the outcomes of operations and relying on the numerosities displayed on the monitor.

### Decoding of arithmetic and hand behaviour

**Figure 7:**
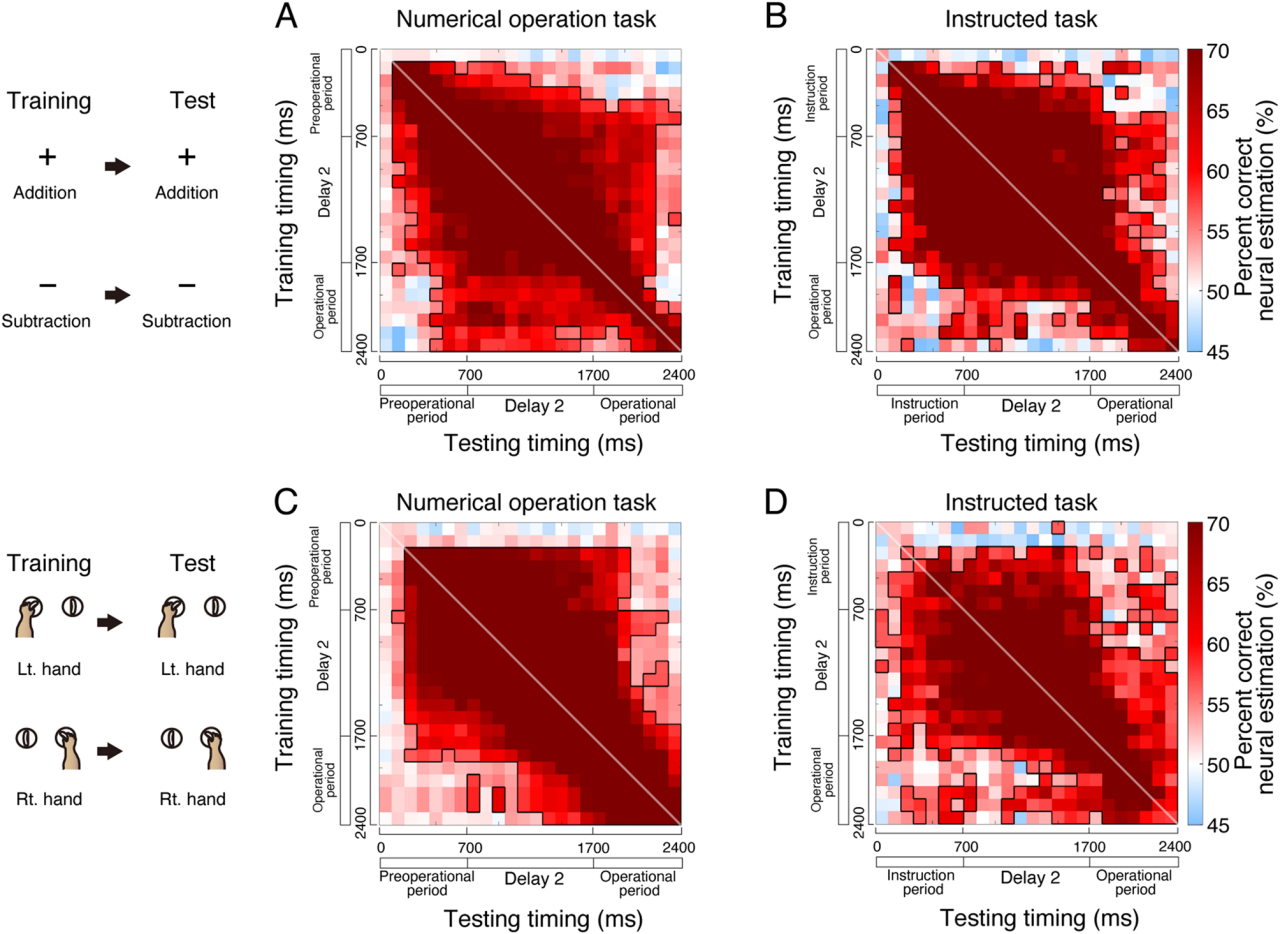
Neural decoding from arithmetic cells. (**A**,**B**) Performance of decoding arithmetic operation assessed using the support vector machine in the numerical operation task (**A**) and the instructed task (**B**). (**C**,**D**) Performance of decoding hand operation using the support vector machine in the numerical operation task (**C**) and the instructed task (**D**). Each row corresponds to a training time while each column corresponds to a testing time (pixel width = 100 ms). Diagonal lines indicate that training and testing occurred simultaneously. Pixels surrounded by bold lines indicate performance significantly higher than chance (permutation test, *P* < 0.05).

Next, we explored the predictive capacity of arithmetic cells in relation to monkey behaviour using support vector machines. We adopted temporal cross-training analysis to assess the stability of encoding^42,43^. We collected data from 72 arithmetic cells during counterbalanced trials for analysis. Employing a tenfold cross-validation approach, we trained on 90% of trials and tested on the remaining 10%. Initially, we predicted the monkey’s addition and subtraction behaviour based on the neural activity of arithmetic cells during the numerical operation task. Our analysis revealed that neural activity within a specific time window (Fig. 7A, along the diagonal line) effectively predicted addition and subtraction behaviour, extending from 100 ms after the presentation of the preoperational numerosity to 700 ms into the operation period (chance rate: 50%). Cross-temporal decoding demonstrated consistent prediction across various time windows, which implies a static coding mechanism (Fig. 7A, off the diagonal line). Comparable results were observed in the instructed task, suggesting that our findings are rooted in arithmetic information rather than the comparison process of larger and smaller values (Fig. 7B). Then, we applied the same approach to predict the monkey’s left and right-hand behaviour based on the activity of arithmetic cells. From 200 ms after the presentation of the preoperational numerosity, we successfully predicted hand usage with the same neural activity timing of arithmetic cells (Fig. 7C,D, along the diagonal line) across both tasks. Temporal cross-training analysis revealed a static coding pattern with a square appearance (Fig. 7C,D, off the diagonal line). Overall, our analysis demonstrated that arithmetic operations (addition or subtraction) and hand usage (left or right) can be reliably predicted by arithmetic cells through a static coding mechanism.

### Cross operation decoding

**Figure 8:**
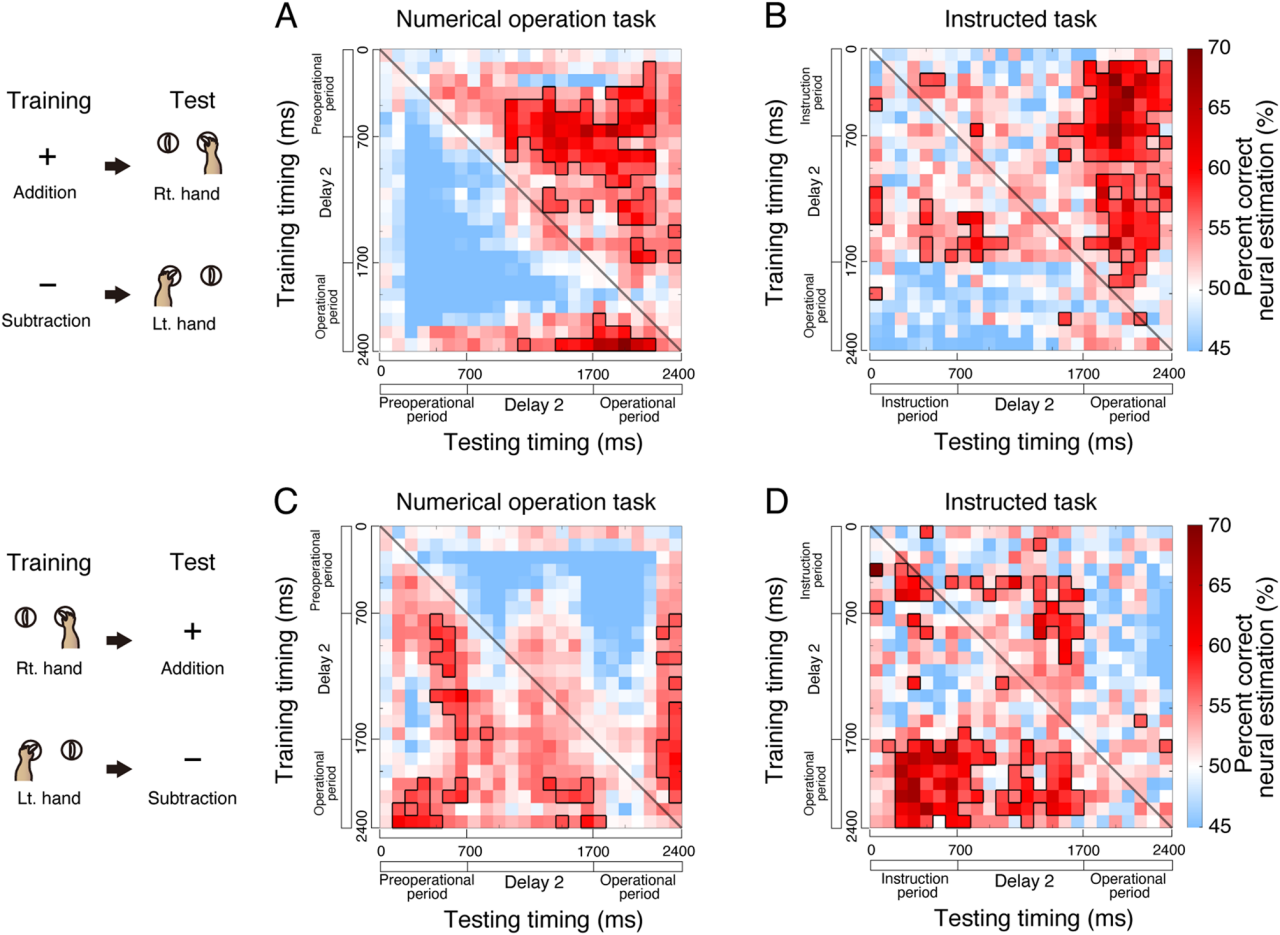
Cross-operational decoding by reusing the classifier. (**A**,**B**) Considering addition as right hand and subtraction as left hand, each classifier trained in Fig. 7A,B was reused in (**A**) and (**B**), respectively. Performance of the decoding hand operation based on the classifier trained to arithmetic in the numerical operation task (**A**) and the instructed task (**B**). (**C**,**D**) Considering right hand as addition and left hand as subtraction, each classifier trained in Fig. 7C,D was reused in (**C**) and (**D**), respectively. Performance of decoding arithmetic operation based on the classifier trained to hand use in the numerical operation task (**C**) and the instructed task (**D**). Diagonal lines indicate that training and testing occurred simultaneously. Pixels surrounded by bold lines indicate performance significantly higher than chance (permutation test, *P* < 0.05).

To investigate the potential overlap between motor commands and arithmetic operations, we conducted cross-operation decoding. Building upon the work of Knops et al.^20^, we explored whether a classifier trained for arithmetic could also be used for hand discrimination, and vice versa. First, we tested whether the same arithmetic classifier, without additional training, could be generalized to classifying hand use. We assigned addition to the right hand and subtraction to the left hand and performed cross-temporal decoding during the numerical operation task. Within the same time window (Fig. 8A, along the diagonal line), the addition classifier could not predict hand use. However, cross-temporal decoding revealed a significant percentage of time windows predicting future hand use (prospective decoding, Fig. 8A, upper right side of the diagonal line: 29.0%, 80 out of 276 windows; binomial test with *P*_chance_ = 0.05, *P* < 0.01). Conversely, the percentage of time windows predicting past hand use was at chance level (retrospective decoding, Fig. 8A, lower left side of the diagonal line: 4.7%, 13 out of 276 windows; binomial test, *P* = 0.63). In the instructed task (Fig. 8B), prospective decoding was again successful (prospective decoding: 31.5%, 87 out of 276 windows; binomial test, *P* < 0.01), suggesting that the process of comparing larger or smaller values did not account for the results. Therefore, the arithmetic classifier could be generalized to predict future hand use, corresponding addition to the right hand and subtraction to the left-hand.

Conversely, we investigated whether the hand use classifier could be generalized to arithmetic classification. Cross-temporal decoding during the numerical operation task (Fig. 8C) demonstrated a significant percentage of time windows predicting past arithmetic operations (retrospective decoding: 12.7%, 35 out of 276 windows; binomial test, *P* < 0.01), but not future arithmetic operations (prospective decoding: 5.8%, 16 out of 276 windows; binomial test, *P* = 0.31). Retrospective decoding was successful in the instructed task (Fig. 8D, retrospective decoding: 29.0%, 80 out of 276 windows; binomial test, *P* < 0.01), ruling out the process of comparing larger or smaller values. Therefore, the hand classifier could generalize to predict past arithmetic operations. Overall, the population activity patterns for arithmetic operations closely resembled those for subsequent hand use when addition was associated with the right-hand and subtraction with the left-hand. Consequently, PMd cells dynamically altered their coding from arithmetic to hand use by reusing analogous population coding features.

## Discussion

We identified cells in the monkey’s PMd that are related to arithmetic operations. Interestingly, many of these arithmetic-related cells transitioned their coding to represent motor components involving hand and step movements. Specifically, we observed that addition and subtraction were preferentially associated with the right-hand and left-hand, respectively, in a simultaneous coding manner. Our decoding analysis demonstrated that classifiers for addition and subtraction could predict the future actions of the right and left-hands, indicating a shared population coding structure over time. These findings suggest that the PMd plays a crucial role in executing arithmetic operations by recruiting the motor system.

Regarding numerical rules, a previous study reported activities related to numerical larger or smaller rules in the PFC of monkeys^44^. Neural activity preceded the presentation of the second operand, suggesting that it was more related to the rule (larger or smaller) rather than the actual process of comparing two numerosities. These activities were stronger in the PMd than in the PFC and cingulate motor areas^30^. Notably, our recording site in the PMd was more ventral compared to that reported in the previous study.

Moreover, independent of notation, arithmetic rule-related neuronal activities were observed in the medial temporal lobe of humans^23^. In this task, participants were instructed to combine the first and second operands according to the given arithmetic rule. Activities related to addition and subtraction were observed irrespective of whether the instructions were conveyed through words or symbols. These rule-selective neurons showed reduced activity after the presentation of the second operand, suggesting that their role was more related to maintaining the arithmetic rule in memory rather than executing the arithmetic operation. The parahippocampal cortex exhibited dynamic coding features, while the hippocampus showed static coding features in decoding analysis, highlighting the different contributions of the medial temporal cortex to arithmetic rule encoding.

In our main task, monkeys implicitly decided whether to add or subtract numerosities following the presentation of preoperational numerosities. Importantly, there were no explicit rule instructions, and arithmetic selectivity emerged after the presentation of the preoperational numerosities. Therefore, neural activity is not the reflection of arithmetic rule instruction. We have elucidated three reasons as to why monkeys are executing arithmetic operations of addition and subtraction. Firstly, behavioural results showed that if the operation leads to undesirable numerical operation (subtraction on an addition required trial or addition on a subtraction required trial), monkeys frequently switched the device and they archived desirable operation (addition or subtraction respectively) in the numerical operation task and the instructed task. The results indicate monkeys have the intention to add or subtract the numerosities. Secondly, device-operation correspondence was transferred through the two tasks, indicating that monkeys have a consistent strategy throughout the two tasks. The strategy is that of how to add or subtract, which is the arithmetic operation itself. Thirdly, we found that the arithmetic-related cells and the neuronal activity were consistent throughout the two tasks. Again, the consistent strategy throughout the both tasks is addition and subtraction. Most of the arithmetic-related cells encoded motor commands for hand movements, suggesting their involvement in numerical manipulation. This study represents the first report of single-cell activities related to arithmetic operations in non-human primates.

In the numerical operation task, the presentation of extreme preoperational numerosities (0, 5 or 6) may force specific choices. When the preoperational numerosity was at its lowest, the only correct choice was to add, with the opposite being true for the highest preoperational numerosities. The accuracy rate was higher when the preoperational numerosity was 6 (Fig. 1C,D), which may reflect the condition. After excluding the 25% of trials with extreme preoperational numerosities from the analysis, we recounted the arithmetic-related cells, which decreased by 12% from 125 to 110 cells. The result suggests that most of arithmetic cells were free from the influence.

This study focused mainly on arithmetic-related cells; however, other cells encoded the number of steps. In the first task, the number of steps was equal to the numerical distance between the target numerosity and the preoperational numerosity, which is essential information for archiving the arithmetic operation. Recent reports have shown that differences in rank (symbolic distance) modulate the spatial selectivity of PMd neurons^31,32^. Future studies should address whether numerical distance is represented in the PMd.

Human brain imaging studies have also identified brain regions associated with arithmetic operations. Bilateral intraparietal and prefrontal areas were activated during both symbolic^15–19^ and nonsymbolic^20,21^ arithmetic tasks, suggesting a non-verbal arithmetic domain^22^. These studies often showed premotor activities during arithmetic tasks, but it was debated whether these activities were purely related to motor processes, such as finger counting, subvocalisation, or button presses, primarily due to limitations in temporal resolution. Our results suggest that the premotor cortex is involved in the transformation from numerical operations to motor execution. Recent resting-state functional connectivity analyses, free from motor components, have revealed arithmetic-related connectivity between the parietal cortex and a broad range of the premotor cortex^21,24,25^. Transcranial magnetic stimulation studies also support this view by demonstrating increased corticospinal excitability in hand muscles during numerical judgment tasks^45^. Consequently, the premotor cortex may represent another non-verbal arithmetic-related domain, warranting further investigation in future studies. We do not rule out the possibility that other upstream brain areas, such as the PFC and the parietal cortex, are involved in arithmetic operations. Rather, we suggest that the premotor cortex is involved in the process of transforming the arithmetic commands produced by these areas into actual motor actions.

Numerous studies have suggested that humans conceptualize numbers in a spatially oriented mental number line, typically running from left to right (the ‘mental number line”)^46,47^. One of the most influential experimental findings supporting this notion is the spatial-numerical association of response codes (SNARC) effect^48^. During parity judgment tasks, individuals tend to respond more quickly with their left-hand for smaller numbers and with their right-hand for larger numbers. Interestingly, this effect was reversed in Iranian readers who read from right to left, suggesting that cultural experiences can modify this number-space mapping.

However, recent evidence has shed light on the existence of a mental number line in non-verbal animals as well^49–52^. In a seminal study, Rugani et al*.* demonstrated that 5-day-old chicks exhibit a left-to-right counting preference^49^. Moreover, they observed that presentations of smaller numerical elements than conditioned numerosity attracted 3-day-old chicks’ responses towards the left panel and vice versa^50^. Adachi also found that chimpanzees responded faster when the number 1 was on the left and 9 was on the right^51^. This alternative perspective suggests that the mental number line may be biologically inherent in the neural systems of various species.

In human studies, the mental number line concept has recently been extended to arithmetic. The interaction between arithmetic and spatial representation (addition-right space and subtraction-left space) has been consistently reported. Mathieu et al*.* found that presenting the second operand to the right of the screen led to faster addition responses, while presenting it to the left led to faster subtraction responses^53^. Patients with left spatial hemi-neglect showed more errors in subtraction than in addition^54^, and there have been rare cases of patients with right visuospatial neglect who had impairments in solving addition but not subtraction^55^. Consistent with these human studies, our research identified neuronal activity indicating that addition is associated with the right-hand, while subtraction is associated with the left-hand. This suggests that there may be a neural substrate underlying the interaction between arithmetic and spatial representation.

Furthermore, Knops et al*.* demonstrated that decisions involving addition and subtraction can be predicted from right and left saccade-related activity in the posterior parietal cortex^20^. This implies that the activation patterns during arithmetic tasks resemble those during saccades, aligning with the concept of cortical recycling. Similarly, in our study, we observed that addition and subtraction decisions could be predicted from right and left hand-related neuronal activity in the monkey PMd. This suggests that the premotor cortex might also serve as another cortical recycling circuit during arithmetic operations. Future studies that investigate whether addition or subtraction decisions can be predicted from right or left-hand-related activity in the premotor cortex of humans would be valuable.

In the present study, we found arithmetic related activity from monkey’s PMd, however there is a limitation for interpretation. Because neuronal data in this study is collected from a single monkey, the current results are not generalized to other monkeys. The involvement of PMd in arithmetic operations should be further explored in future studies.

## Methods

### Behavioural tasks and stimuli

We trained two male *Macaca fuscata* monkeys, monkey N (weight = 7.9 kg) and K (weight = 6.5 kg), to perform two types of numerical operation task using specific devices (Fig. 1A,G). Both monkeys were approximately 8 years old. All animal care and research procedures were conducted in strict accordance with the Guiding Principles for the Care and Use of Laboratory Animals set forth by the US National Institutes of Health. They are also in accordance with ARRIVE guidelines. Our research was approved by the Institutional Animal Care and Use Committee of Tohoku University. We used C++ Builder software (Borland, Austin, TX, USA) to run the task.

The first task, known as the “numerical operation task”, aimed to precisely match the numerosity of visual objects currently displayed with the numerosity of target objects initially presented on the screen at the beginning of each trial. The monkeys operated the devices to either increase or decrease the number of displayed circles incrementally. The trial began with the monkey gazing at a fixation point on the screen for 800 ms (the “fixation period”). Subsequently, 1–4 white circles appeared within a red square frame for 700 ms, representing the target numerosity. Following a 1000 ms delay (Delay 1 period), during which a grey square frame was displayed, 0–6 circles appeared as the “preoperational numerosity” within a blue square frame for 700 ms. After a second delay of 1000 ms (Delay 2 period), during which a grey square frame with a black screen was shown, the preoperational numerosity reappeared, accompanied by a tone signal. Upon the tone signal, the monkeys were prompted to either add or subtract the preoperational numerosity to match the target numerosity. The monkeys had to maintain their gaze on a fixation point (a red circle, 1.4° in visual angle) from the fixation point until the Go signal. There were 19 combinations of target numerosities and preoperational numerosities presented randomly. The monkeys manipulated the left device in a clockwise rotation or the right device in a counter-clockwise rotation to adjust the number of visual objects displayed on the monitor screen. Under Rule 1, using the left device increased the numerosity by one, while using the right device decreased it by one. Under Rule 2, the effects of device use were reversed. Each rule was blocked for several trials, so monkeys were required to perform operations under the current rules with the goal of numerical matching. If they stopped device use for 1.5 s, the displayed numerosity was considered their decision (chosen numerosity). The monkeys were allowed to use either device freely at their own pace as long as they did not stay for 1.5 s. Rewards were delivered based on the chosen numerosity, and correct matching was rewarded with a blue square and a juice. There was no time limit or step limit for device use, and they could select numerosities ranging from 0 to 6. If the preoperational numerosity already matched the target numerosity, the monkeys may maintain the preoperational numerosity for 1.5 s. Incorrect matching or the use of both devices simultaneously resulted in a red square appearing on the screen (an error signal), and a new trial began. The inter-trial interval was set at 3 s, regardless of whether the matching was correct or incorrect. In this task, chance performance was considered 25%.

In the second task, referred to as the “Instructed task”, the monkey was required to execute a specified operation as instructed. The task followed the same timing schedule as the first task, but with the following differences:

1. After the fixation period, a red square appeared for 700 ms, signalling the second task.
2. Following Delay 1, visual instructions for the subsequent operation were presented within a blue square frame. If the instruction showed a “+”, exceeding the preoperational numerosity was considered correct. If the instruction displayed a “–”, having a numerosity less than the preoperational numerosity was deemed correct.
3. There were three possible preoperational numerosities (1–3) for each type of instruction (either + or −), resulting in a total of six possible combinations.

For example, if the instruction was “+” and the preoperational numerosity was 2, numerosities of 3–6 were considered correct (however, the monkeys selected numerosity 3 in most of trials; see Fig. 1J). Monkeys were allowed to use both devices freely and the reward was fully contingent on the chosen numerosity. Therefore, the instructions provided no information about the hand to use. The monkeys were required to perform the instructed operation on the preoperational numerosity according to the current rule (Rule 1 or Rule 2).

The target and preoperational numerosities were displayed within 6° × 6° red and blue square frames, respectively. Grey square frames indicated the delay periods. To ensure that cells responded to numerosity rather than low-level visual features, we utilized one standard signal and one control signal, with the same circumference and linear properties, in 50% of the trials^10,11^. Physical appearance was counterbalanced across sets with regard to area, density, and configuration as a function of numerosity (Fig. S1Supplemental information). This design helped control for potential low-level visual cues.

### Rule switch and task switch

During the initial training phase, we introduced the monkeys to the numerical operation task under Rule 1. After the success rate reached the criterion of 70%, we introduced Rule 2. When the success rate reached the criterion, Rule 1 was reintroduced. Both rules were applied every other day. Finally, the rule was switched every 50 trials across 1000 daily trials. The rule switch was not explicitly indicated by external signals; the monkeys had to determine which rule was in effect through trial and error. This behavioural paradigm was described in detail previously^41^. Data related to the SNARC-like effect (Fig. S6) were collected during three sessions under these conditions.

Following this initial behavioural testing phase, we introduced the instructed task under Rule 1. Subsequently, we introduced Rule 2 and then switched the rules in the same manner as for the numerical operation task. Subsequently, the monkeys were trained on both tasks in separate sessions. We continued training until the monkeys reached a performance plateau. Once the performance curve plateau was achieved, we introduced a “task switch”. In this setup, the numerical operation task and the instructed task were organized into blocks and alternately switched. Each block consisted of 25 trials, and the task switch occurred without any explicit instruction. Simultaneously, we introduced a “numerical operation rule switch” at the point of the task switch. Here, the rules for numerical operation (Rule 1 and Rule 2) were blocked and alternately switched every 100 trials without any specific instruction. The data related to the behavioural strategy (Fig. S2) and the task switch (Fig. S3) were collected under these conditions.

During the recording sessions, both the numerical operation task and the instructed task were presented randomly and the rule switch was introduced approximately every 250 trials. We collected main behavioural data (Fig. 1) during these recording sessions.

### Surgical and recording methods

We employed conventional electrophysiological methods to conduct in vivo single-cell recordings^26^ from the PMd in the left hemisphere. After completing the behavioural training, we performed aseptic surgery under pentobarbital sodium anaesthesia (30 mg/kg, intramuscular) with atropine sulphate. An acrylic recording chamber was attached to the skull of the monkey. To prevent postsurgical infection and pain, antibiotics and analgesics were used. Unfortunately, we encountered a huge earthquake, which prevented us from maintaining the health of a monkey and the experimental facilities. For this reason, neural recordings were performed from Monkey N only.

Prior to the recording sessions, we localized PMd based on previously established physiological criteria^56^ (intracortical microstimulation effects). We also identified the cortical sulci and recording sites through magnetic resonance imaging. The accuracy of these sites was later confirmed through histological examination of brain sections stained with Klüver–Barrera. To monitor eye position, we used an infrared corneal reflection monitoring system (Millennium G200; Matrox, Dorval, Canada). Neural activity was recorded using an eight-channel electrode (Multitrode; Thomas Recording, Giessen, Germany) inserted through the dura mater using a hydraulic microdrive (MO-81; Narishige, Tokyo, Japan). The neural signals were amplified by a Plexon headstage (Plexon, Dallas, Tx, USA; gain = 20×) and a preamplifier (gain = 50×) and bandpass filtered between 150 Hz and 8 kHz (OmniPlex system; Plexon). Data were collected using a multichannel acquisition processor (MAP; Plexon). The activity of single units was isolated offline using spike-sorting software (Offline Sorter; Plexon). Statistical analyses were performed using MATLAB (MathWorks, Natick, MA, USA).

### Data analysis

Our database included cells from which activity was recorded during more than two blocks of numerical operation rules in both tasks. Only the responses for correct trials with the minimum number of steps were analyzed. Initially, we focused on the cells that exhibited modulation in their activity during the preoperational period in the numerical operation task. To assess how various factors related to numerical operations affected cell activity, we conducted linear regression analysis in 100 ms time bins (0–700 ms following the onset of the preoperational numerosity). The following regression model was used:

$${\text{firing}}\;{\text{rate }} = \upbeta_{0} + \upbeta_{1} \times \left( {{\text{arithmetic}}} \right) + \upbeta_{2} \times \left( {{\text{hand}}} \right) + \upbeta_{3} \times \left( {{\text{step}}} \right) + \upbeta_{4} \times \left( {{\text{stimulus}}\;{\text{type}}} \right)$$

where β_0_ is the intercept and β_1_, β_2_, β_3_, and β_4_ are the regression coefficients.

The first factor we considered was the arithmetic operation, specifically whether the required operation was addition or subtraction. The second factor was related to hand movement, distinguishing between left-hand and right-hand actions. The third factor was step, indicating whether the operation involved a single step or two steps. Finally, the fourth factor was stimulus type, classifying whether the stimulus was a normal stimulus or a control stimulus. We represented these factors as dummy variables in linear regression analysis. In each case, we calculated the probability that the coefficient equalled zero. If this probability was < 0.01, we deemed the respective factor as significant. Consequently, we extracted cellular activities that exhibited variation based on arithmetic, hand movement, and step, irrespective of stimulus type, within each time window.

To mitigate the possibility that the observed arithmetic-related activities in the numerical operation task were a reflection of numerical larger or smaller processes, we conducted a second filter using linear regression analysis. For cells that demonstrated arithmetic-related activities, we scrutinized their responses during the instructed task using the following equation:

$${\text{firing}}\;{\text{rate }} = \upbeta_{0} + \upbeta_{5} \times \left( {{\text{instruction}}} \right)$$

where β_5_ is the regression coefficient, whether instructed operation was addition or subtraction was analysed during the instruction period (100 ms time bins, 0–700 ms following the onset of the instruction). If the cells varied their activities according to the instruction during any one of 100 ms time windows, the activities of the cells in the numerical operation task were defined as being related to arithmetic.

Following this procedure, we collected task-related information during the preoperational periods for each PMd cell and meticulously examined their coding history. If a cell exhibited encoding of factors related to arithmetic, hand movement, or step (excluding stimulus type) during any of the 100 ms bins, the factor was recorded in the coding history. Based on coding history, we classified the cells as arithmetic-related, hand-related, and step-related. These categories could also overlap (Fig. 2F). Our primary focus was on the cells that met the aforementioned criteria. Subsequently, we further investigated their activities during two subsequent periods: Delay 2 period (1000 ms following the conclusion of the preoperational numerosity presentation) and Operation period (700 ms following the Go signal) (Fig. 4).

### Neural decoding

The arithmetic-related cells that met the criteria (cells recorded at least 10 trials for a combination of each of four types of target numerosity: two types of arithmetic [addition and subtraction] and two types of hand use [left vs. right] making up 160 trials) were entered into a linear support vector machine classifier using the LIBSVM library. The activities of the arithmetic-related cells during the Concern period (from the presentation of preoperational numerosity to 700 ms following the Go signal) were split by a 100 ms time window, preparing 24 windows in all.

In simulations, we adapted tenfold cross-validation in which a linear support vector machine was trained to randomly select nine-tenths of trials and performances were evaluated on the remaining trial. The procedure was repeated 10 times so that every split was tested once; the average rate of correct classifications was calculated. To assess the significance of the classification performance, arithmetic labels and hand labels were shuffled and the average performance was calculated 1000 times. This provided a null-hypothesis distribution of performance with an expected mean accuracy of 50%. A classifier accuracy exceeding the value of the 50th highest rank of distribution was considered significant (permutation test, *P* < 0.05).

For temporal cross-training analysis, each training set of 24 time windows was paired with a corresponding testing set of 24 time windows, for a total of 576 time windows. Temporal cross-training analysis considered three types of decoding: the same decoding for both the training and testing timing (24 time windows), prospective decoding in which the training timing preceded the testing timing (276 time windows); and retrospective decoding in which the testing timing was followed by the training timing (276 time windows). We evaluated the three types of decoding using a binomial test with a chance probability for each pixel set to p = 0.05.

### Supplementary Information

Supplementary Figures.

## Data Availability

All data that support the findings of this study are presented in the paper and the Supplementary information.
