# Supplementary material for: Recruitment of the premotor cortex during arithmetic operations by the monkey
Source: Sci Rep. 2024 Mar 28;14:6450. doi: 10.1038/s41598-024-56755-2 (PMC10978941; doi:10.1038/s41598-024-56755-2)
Supplement: Supplementary file 1 — Supplementary Figures. [file 41598_2024_56755_MOESM1_ESM.pdf]

Title: Recruitment of the premotor cortex during arithmetic operations by the monkey

Authors: Sumito. Okuyama, Toshinobu. Kuki, Hajime. Mushiake

Supplementary Figures:

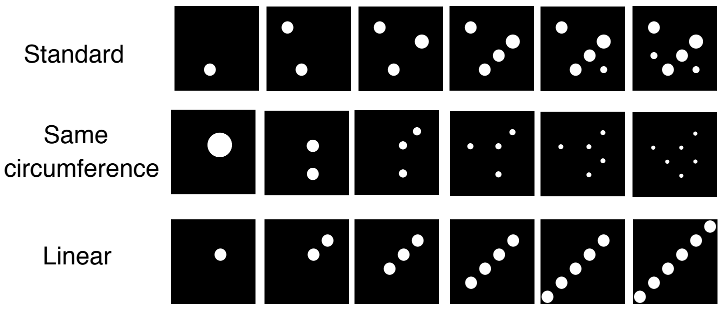

Figure S1 I Examples of three versions of stimuli. Stimuli used in this study included standard displays and versions of displays with the same circumference and linear properties.

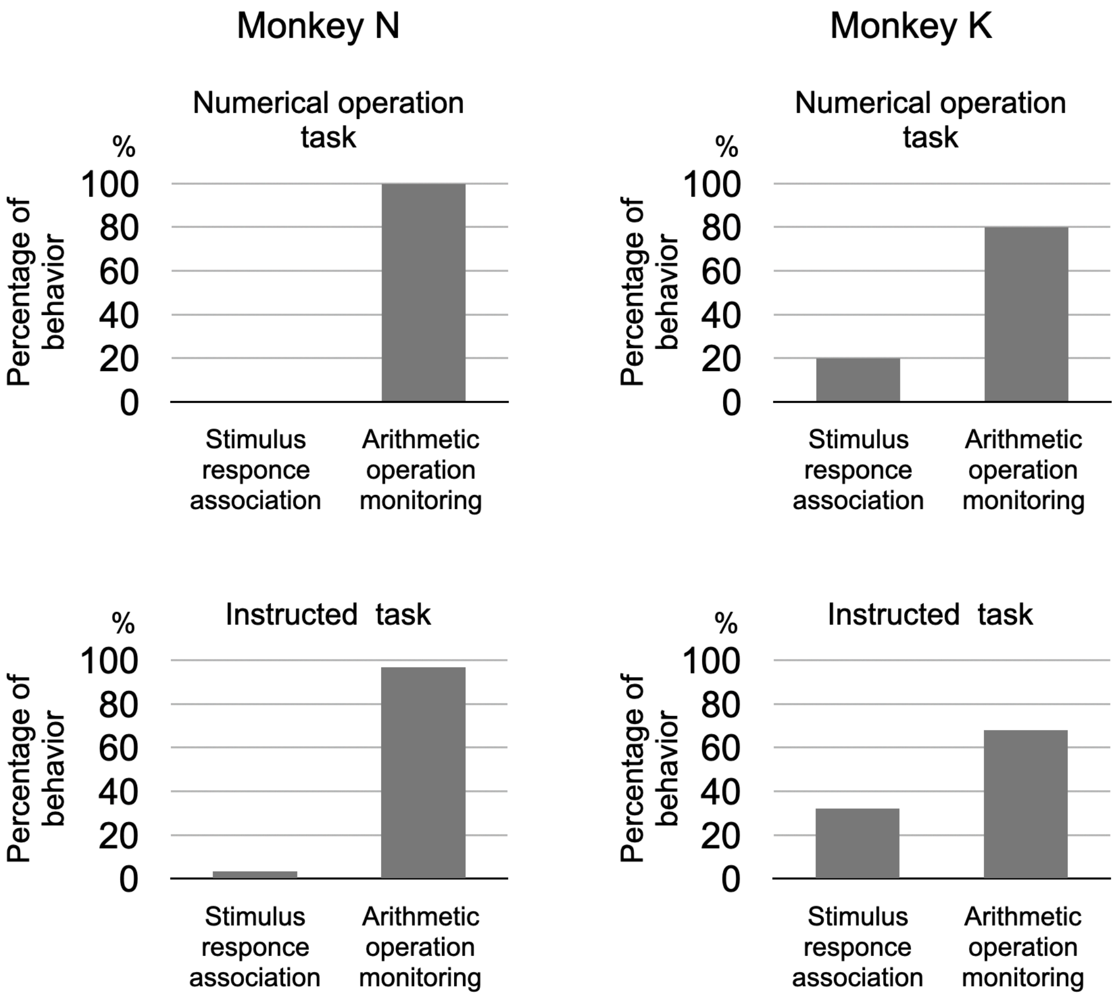

Figure S2 I Percentage of correct operations following an inappropriate operation in the first step. After an inappropriate operation in the first step, we determined whether the monkeys exhibited switching behaviour in the absence of reward contingency ("arithmetic operation monitoring"). The monkeys used the arithmetic operation monitoring strategy more frequently than the stimulus-response association strategy, both in the numerical operation task (Monkey N, 100%, $P < 0.01$, binomial test with $P_{chance} = 0.5$; Monkey K, 80%, $P < 0.01$) and the instructed task (Monkey N, 96.7%, $P < 0.01$; Monkey K, 68%, $P < 0.01$).

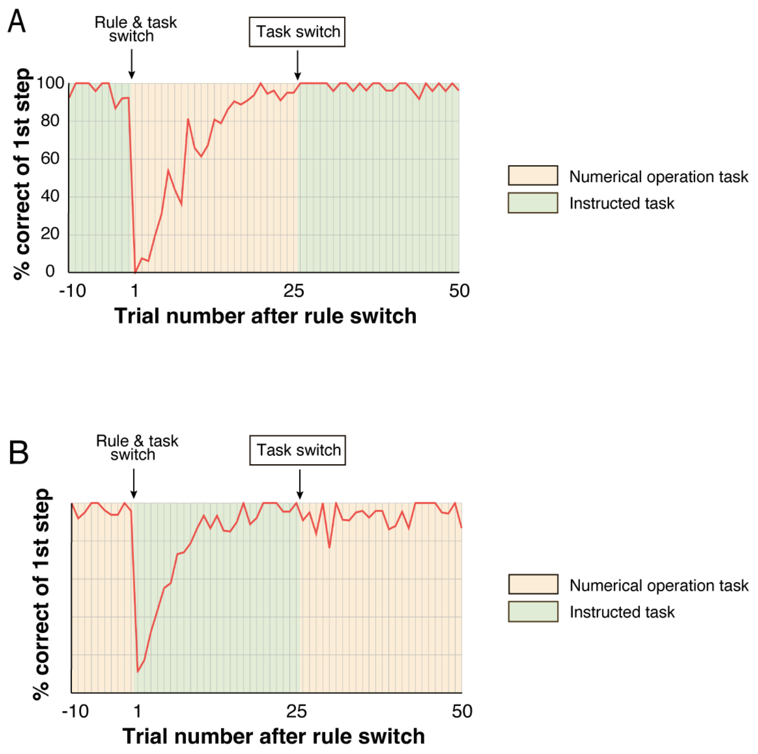

Figure S3 I Average percentage of correct operations in the first step by both monkeys during rule switch with or without a task switch. (A)Task switch from the numerical operation task to the instructed task did not affect performance during the first step (McNemar test, $P = 1$). (B)Task switch from the instructed task to the numerical operation task did not influence performance during the first step (McNemar test, $P = 0.25$).

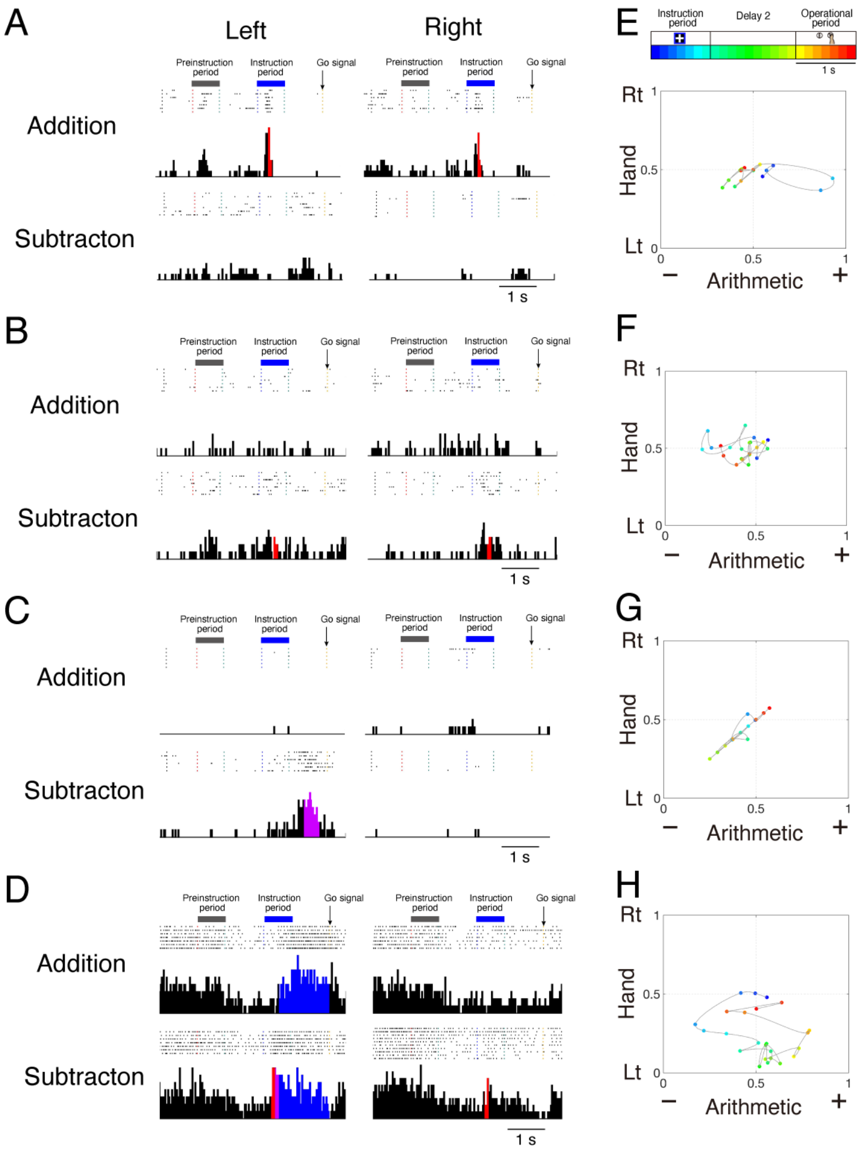

Figure S4 I Examples of arithmetic-related cell activity during the Instructed task. Raster displays and peri-event histograms illustrating the cellular activity for addition (**A**), subtraction (**B**), subtraction by left hand (**C**), and transition from subtraction to the left hand (**D**) (Results from the same cell shown in Figure 2A, 2B, 3A and 3B, respectively). (**E**) ROC values for each operation (arithmetic and hand) from the same cell shown in (**A**), representing the addition-related trajectory. (**F**) Subtraction-related trajectory from the cell shown in (**B**). (**G**) Combination of the arithmetic- and hand-related trajectory from the cell shown in (**C**). (**H**) Trajectory from the cell shown in (**D**), transitioning from arithmetic to hand.

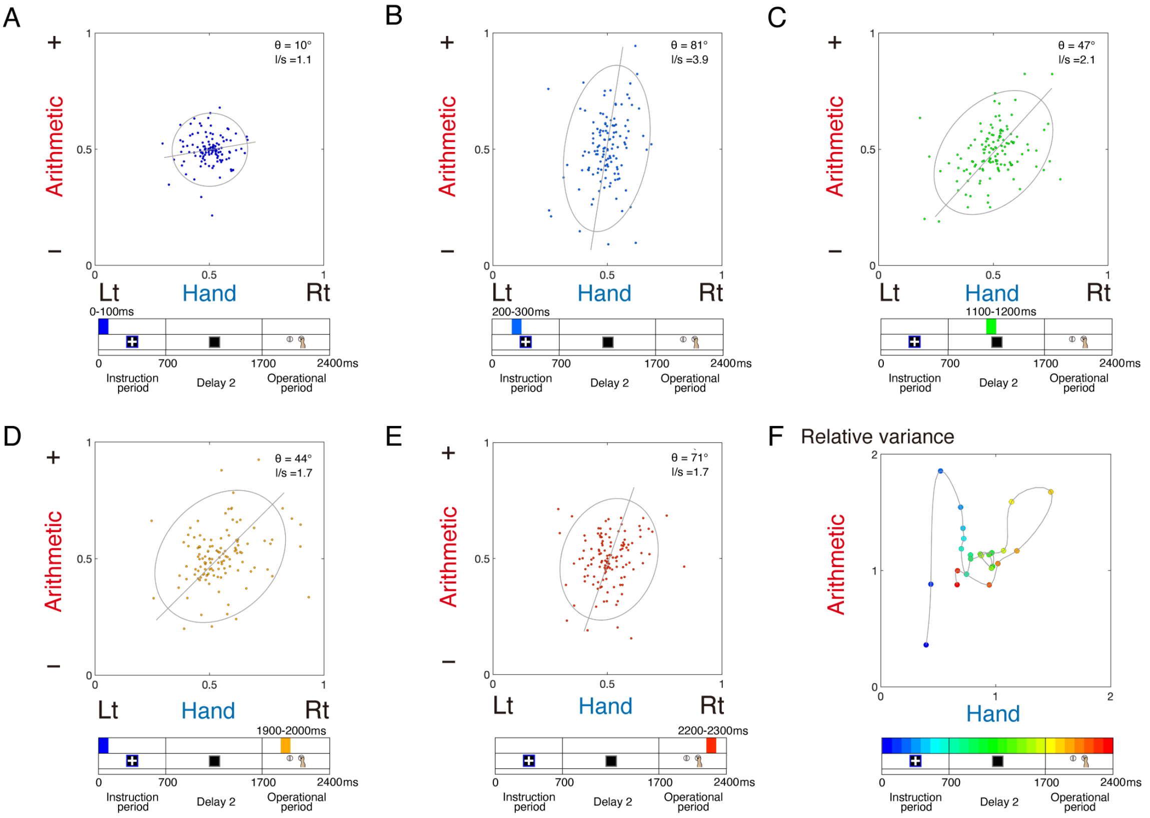

Figure S5 l Dynamic response by a population of arithmetic cells in the instructed task. ROC analysis for each operation (arithmetic and hand) in 125 arithmetic-related cells. The analysis was performed for the following time windows: (**A**) 0-100 ms, (**B**) 200-300 ms, (**C**) 1100-1200 ms, (**D**) 1900-2000 ms, and (**E**) 2200-2300 ms after the onset of instruction. Two-dimensional Gaussian fit was performed for the data. Ellipses represent 95% confidence contours. θ, angle of ellipse's long axis relative to axis of arithmetic or hand; l/s: ratio of length of the long to short axis. (**F**) Relative variances of each ROC value, calculated by normalizing to the average variance throughout the relevant period, are plotted.

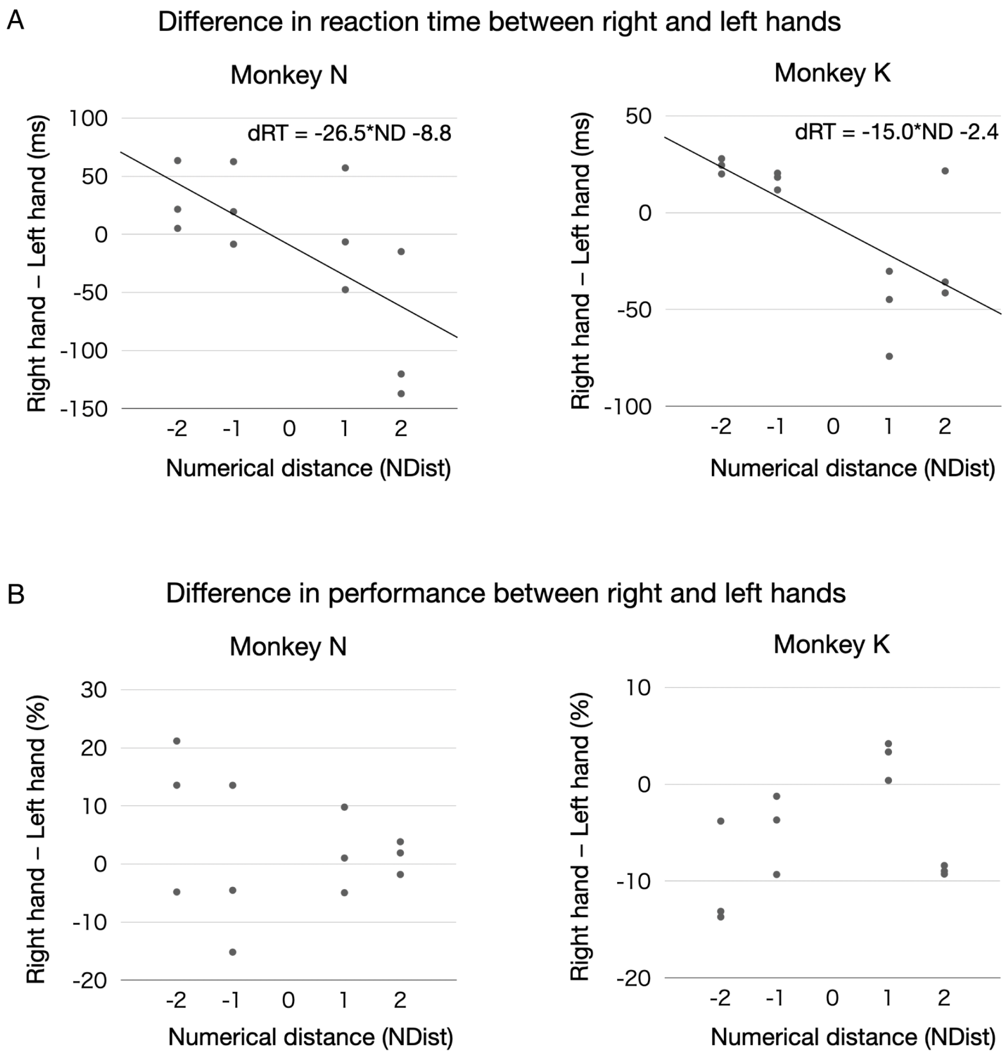

Figure S6 I SNARC-like effect. Gray dots indicate the differences in (**A**) reaction time (dRT = RT_{right} – RT_{left}) and (**B**) accuracy rate (dAR = AR_{right} – AR_{left}) between the right and left-hands in the numerical operation task as a function of numerical distance (NDist = Target numerosity – Preoperational numerosity). A positive value of NDist corresponds to addition trials, and a negative value corresponds to subtraction trials. (**A**) dRT was negatively correlated with NDist (Monkey N, $t[10] = 2.85$, $P < 0.05$; Monkey K, $t[10] = 3.85$, $P < 0.01$). The results indicate that the left-hand response was faster in subtraction trials than in addition trials, whereas the right-hand response was faster in addition trials than in subtraction trials. (**B**) dAR was not correlated with NDist (Monkey N, $t[10] = 0.70$, $P = 0.50$; Monkey K, $t[10] = 0.90$, $P = 0.39$).
